# Prevention of anesthesia-induced injection pain of propofol in pediatric anesthesia

**DOI:** 10.12669/pjms.333.12026

**Published:** 2017

**Authors:** Dabin Cheng, Lu Liu, Zheng Hu

**Affiliations:** 1 Dabin Cheng, Department of Anesthesiology, Children’s Hospital of Nanjing Medical University, No. 72 Guangzhou Road, Nanjing 210008, Jiangsu Province, China; 2 Lu Liu, Department of Anesthesiology, Children’s Hospital of Nanjing Medical University, No. 72 Guangzhou Road, Nanjing 210008, Jiangsu Province, China; 3 Zheng Hu, Department of Anesthesiology, Children’s Hospital of Nanjing Medical University, No. 72 Guangzhou Road, Nanjing 210008, Jiangsu Province, China

**Keywords:** Injection pain, Lidocaine, Ketamine, Propofol

## Abstract

**Objective:**

Propofol is a new anesthetic agent in clinical practice, but randomized double-blinded prospective studies on its role in pediatric anesthesia remain limited. We aimed to compare the preventive effects of pre-injected lidocaine or ketamine and its pre-mixture on the anesthesia-induced injection pain of propofol using a randomized double-blinded prospective method, and to compare the outcomes with those of medium-/long-chain propofol (M/LCT).

**Methods:**

A total of 360 pediatric patients (aged 5-12 years old) who received elective surgery were randomly divided into six groups (n= 60) as follows. S group: control group; L group: lidocaine group; L + P group: lidocaine + propofol group; K group: ketamine group; K + P group: ketamine + propofol group; M group: M/LCT group. After the drug fluid completely entered the cubital vein, the venous access was closed. During propofol injection, the injection pain was scored using the VRS 4-point scale. Meanwhile, the heart rates before and during injection were recorded, the adverse reactions during and after injection were observed, and the incidence rate and degree of pain were evaluated.

**Results:**

The VRS 4-point scale showed that the incidence rates of injection pain of S group, L group, L + P group, K group, K + P group and M group were 78.3%, 66.67%, 51.66%, 43.33%, 48.33% and 45% respectively. The incidence rates of injection pain of all experimental groups were significantly lower than that of S group (P<0.01). The incidence rates of injection pain of L + P group, K group, K + P group and M group were significantly lower than that of L group (P<0.05). The differences among the other groups were not statistically significant.

**Conclusions:**

Intravenous pre-injection of lidocaine, ketamine or those mixed with propofol can all significantly reduce the incidence rate of injection pain of propofol.

## INTRODUCTION

Propofol is a novel ultra-short-acting intravenous anesthetic agent with rapid effects,^1^ the main adverse reaction of which is injection pain. Obvious pain can be caused by peripheral intravenous injection.^2^ Propofol injection pain ranks 7th among the 33 major problems of clinical concern, with the incidence rates of 28%-90%.^3^

Among pediatric patients, the injection pain of propofol is an intractable problem. To solve the problem, anesthesiologists have used a number of methods, such as local anesthesia and cooling of drug fluid. In most cases, propofol injection is mixed with lidocaine.^4^ A meta-analysis showed that intravenous administration of 0.5 mg/kg lidocaine after tying a rubber tourniquet on the forearm 30-120 s before the injection of propofol relieved the pain of about 60% of patients.^5^ However, even using a variety of methods, propofol injection pain still cannot be completely prevented, with the incidence rates as high as 32%-48%.^6^

Currently, the commercially available formulation of dosage form is prepared by dissolving 1% propofol in 10% soybean oil emulsion. The emulsion contains complex ingredients, so the adaptability to intravenous infusion depends on many factors. The instability of emulsion itself may be one of the causes for injection pain.^7^ Medium-/long-chain propofol (M/LCT) can increase the lipid solubility and decrease the aqueous phase concentration of propofol by using medium-/long-chain fatty acids as solvents, thereby reducing the incidence rate of injection pain. The preparations of 10% medium-/long-chain triglycerides can decrease the aqueous phase concentration of propofol to (14.0 ± 0.5) mg/L, with its injection pain reduced by 25% compared with that of long-chain triglyceride only [(18.6 ± 0.6) mg/L]^8^ in the absence of other preventive measures. The incidence rate and degree of pain of the latter strategy remained almost unchanged even when 10 mg lidocaine was pre-injected. Compared with long-chain propofol, the injection pain of M/LCT can be markedly reduced,^9^ but the sample size is small. Besides, related studies on pediatric injection pain remain scarce.

Thereby motivated, we designed a randomized double-blinded prospective study to observe the incidence rate and degree of injection pain of M/LCT during induction of pediatric anesthesia using the VRS 4-point scale. The results were compared with those of injection of long-chain propofol, pre-injection of 0.5 mg/kg lidocaine or 0.2 mg/kg ketamine before propofol injection, and injection of 180 mg propofol mixed with 40 mg lidocaine or 16 mg ketamine. We aimed to provide evidence for the clinical application of M/LCT by evaluating the preventive effects of these regimens on the injection pain and adverse reactions of propofol.

## METHODS

### Inclusion criteria

With approval by the ethics committee of our hospital and written consent of guardians; with preoperative scores classified by the American Society of Anesthesiologists (ASA) standards^10^ as Grade I to II; aged 5-12 years old.

### Exclusion criteria

With body mass index >30 or <18; children allergic to local anesthetics, lipid drugs, propofol or ketamine; with asthma, neurological dysfunction or mental disorder; with preoperative ASA classification of Grade III-IV and emergency admission; with liver or kidney dysfunction; with phlebitis; with failure in venipuncture over twice; non-compliant children who cried and screamed.

### Grouping

The eligible 360 patients who received elective surgery under general anesthesia were randomly divided into six groups (n=60) as follows. S group: The patients were given 2 ml of 0.9% sodium chloride (Sinopharm Group Co., Ltd., Beijing, China) and then a mixture of propofol (Beijing North Institute of Biological Technology, China) and 0.9% sodium chloride 30 s later; L group: the patients were given 0.5 mg/kg lidocaine (Beijing North Institute of Biological Technology, China) and then a mixture of propofol and 0.9% sodium chloride 30 s later; L + P group: the patients were given 2 ml of 0.9% sodium chloride and then a mixture of propofol and lidocaine 30 s later; K group: the patients were given 0.2 mg/kg ketamine (Beijing North Institute of Biological Technology, China) and then a mixture of propofol and 0.9% sodium chloride 30s later; K + P group: the patients were given 2 ml of 0.9% sodium chloride and then a mixture of propofol and ketamine 30 s later; M group: the patients were given 2 ml of 0.9% sodium chloride and then a mixture of propofol M/LCT (Beijing North Institute of Biological Technology, China) and 0.9% sodium chloride 30 s later.

### Anesthetic methods

All patients underwent routine preoperative fasting, without any premedication. A 24G intravenous catheter was placed and connected with a t-tube that was thereafter rinsed with lactated Ringer’s solution (Beijing SanYao Science & Technology Development Co., Ltd., China). After the children stopped crying and screaming, they were randomly divided when the liquid infusion was unobstructed. Electrocardiogram, noninvasive blood pressure, saturation of pulse oximetry and end-tidal carbon dioxide partial pressure were routinely monitored (ABI PE-Applied Biosystem, Foster, CA, USA). A 2 ml syringe with 2 ml of drug was connected onto one end of the t-tube which was opened after the children became calm. Then the drug was injected at a speed of 1 ml/6 s. After the drug entered the cubital vein, the infusion channel was closed. Thirty seconds later, anesthesia induction was conducted by intravenous injection of 2.5 mg/kg propofol at a speed of 1 ml/6 s using a constant-speed pump. After consciousness disappeared or breathing stopped, the children were subjected to 100% oxygen-assisted ventilation, followed by injection of 2 μg/kg fentanyl (Novartis Pharma Schweiz AG, Oslo, Switzerland) and 0.2 mg/kg cis-atracurium besylate (Novartis Pharma Schweiz AG, Oslo, Switzerland) successively. After tracheal intubation was completed, the patients were subjected to inhalation anesthesia and inserted by laryngeal mask, and spontaneous breathing was maintained during surgery.

### Observation indices

Throughout propofol infusion, the facial expressions, language responses, limb movements, arm retraction and crying of the patients were closely observed by an assistant, and their injection pain was scored with the VRS 4-point scale.^11^

With the most severe pain scored as 4 points, 0 point represents no pain, and 1-3 points all suggest pain occurs. The degrees of injection pain were classified as mild, moderate and severe, and the incidence rate of pain in each group was calculated. The heart rates before and during injection as well as the side effects of propofol, such as mental disorder, nausea and vomiting, diplopia, cardiac disorder and allergy, were recorded. After surgery, the patients were followed up for the pain and skin conditions of the venipuncture site to observe whether there were symptoms of phlebitis (e.g. redness and swelling).

### Statistical analysis

All data were analyzed by SPSS16.0. The categorical data were expressed as mean ± standard deviation. Inter-group comparisons were performed by one-way analysis of variance, and intra-group comparisons were conducted by repeated measures analysis of variance data. The numerical data were subjected to Chi-square test, and the ordinal data were subjected to rank sum test. The incidence rates of pain were compared by Chi-square test, and degrees of pain were compared by rank sum test. P<0.05 was considered statistically significant.

## RESULTS

### Baseline clinical data

The genders, ages, body weights, heights and ASA grades of all children were similar (P>0.05) (Table-I).

**Table-I:** Baseline clinical data (n=60).

*Item*	*Age (year)*	*Body weight (kg)*	*Height (cm)*	*ASA grade (I/II)*	*Gender (male/female)*
S group	6.43±1.68	21.38±4.17	113.55±8.97	40/20	38/22
L group	6.36±1.77	23.36±5.77	111.77±9.78	41/19	39/21
L + P group	6.72±1.35	21.68±6.63	103.98±7.19	44/16	36/24
K group	6.68±1.57	21.36±7.39	110.36±9.73	34/6	40/20
K + P group	7.13±1.68	23.55±6.31	113.58±8.99	33/7	38/22
M group	7.15±1.98	24.17±4.99	113.77±10.93	33/7	36/24
F value	1.351	0.779	0.636	1.13	2.797
P value	0.298	0.398	0.798	10.993	0.756

### Incidence rates of propofol injection pain

The incidence rates of injection pain of S group, L group, L + P group, K group, K + P group and M group were 78.3%, 66.67%, 51.66%, 43.33%, 48.33% and 45% respectively. The incidence rates of all experimental groups were significantly lower than that of S group (P<0.01). Besides, the incidence rates of L + P group, K group, K + P group and M group were significantly lower than that of L group (P<0.05). The differences among the other groups were not statistically significant (P>0.05).

### Degrees of propofol injection pain

The degrees of propofol injection pain were evaluated by the VRS 4-point scale (Table-II), and inter-group comparisons were conducted with rank sum test. All the experimental groups had significantly lower degrees of injection pain than that of the control group, and there were significant inter-group differences (P<0.01).

**Table-II:** Degrees of injection pain (case, n=60).

*Group*	*Painless*	*Mild pain*	*Moderate pain*	*Severe pain*
S group	13	15	25	7
L group	20	19	20	1
L + P group	29	18	12	1
K group	24	20	16	0
K + P group	21	20	17	2
M group	23	17	19	1

### Heart rates before and after injection

The heart rates of all groups, which were similar before propofol injection (P>0.05), were significantly elevated after it (P<0.05). After injection, S group had significantly higher heart rate than those of L + P, K + P and M groups (P<0.05). The heart rate of L group was significantly higher than those of L + P and K + P groups (P<0.05). The heart rates of other groups were not significantly different (P>0.05) (Table-III).

**Table-III:** Heart rates before and after injection.

*Item*	*Before injection*	*After injection*
S group	92.63±10.33	116.43±12.46*
L group	94.78±12.33	116.47±12.49*#Δ
L + P group	95.77±10.56	105.35±9.47*
K group	97.35±11.28	109.38±12.76*
K + P group	93.56±13.46	105.33±13.47*#
M group	94.99±10.37	106.48±10.47*#Δ

Compared with heart rate before injection,

* P<0.05; compared with S group,

# P<0.05; compared with L group,

Δ P<0.05.

### Adverse reactions after injection

After propofol injection, there were no significant differences between the adverse reactions of all groups (P>0.05) (Table-IV).

**Table-IV:** Adverse reactions after injection (n=60).

*Group*	*Mental disorder*	*Nausea and vomiting*	*Diplopia*	*Cardiac disorder*	*Allergy*	*Phlebitis*
S group	0	1	5	0	0	0
L group	0	2	1	0	0	0
L + P group	0	2	1	0	0	0
K group	2	4	0	0	0	2
K + P group	2	4	0	0	0	0
M group	0	1	0	0	0	0

## DISCUSSION

As an innovation of anesthesia, propofol has now been widely used due to rapid action and short functioning time. However, it mainly suffers from injection pain,^12^ as an inevitable issue of which even adults are afraid.^13^ With elusive mechanisms, the injection pain of propofol has mainly been attributed to the pain-inducing effect originating from the contact between aqueous phase of emulsion and free nerve endings^14^ or the delaying effect based on bradykinin produced by the activated kinin cascade system.^15^ Bradykinin leads to local phlebectasia and increases vascular wall permeability, so propofol can penetrate the vascular wall to contact with more free nerve endings, thus aggravating the injection pain.^16^

The incidence rates of propofol injection pain in adults range from 28% to 90%, while those of children range between 30% and 90%. Mixing propofol with 1% lidocaine in inducing the anesthesia of preschoolers can dramatically reduce the injection pain (59% vs 22.5%).^17^ Lidocaine may be able to stabilize the kinin cascade induced by propofol. Nevertheless, lidocaine usually destabilizes propofol injection to produce lipid droplets, and those larger than 5 μm may result in fat embolism.^18^

In this study, S and L groups were more prone to propofol injection pain than other groups, probably because the patients did not receive premedication and fine puncture needles were used for veins. M/LCT, as a novel preparation that dissolves propofol in 10% medium-/long-chain triglyceride, can mitigate the injection pain by decreasing the free concentration of propofol in aqueous phase. It works for both adults and children.

The incidence rate of pain after M/LCT injection, which was significantly lower than that after normal saline treatment (47.5% vs 95%), was slightly higher (0.2%) than that after pre-mixing lidocaine (P>0.05). As the receptor antagonist for N-methyl-D-aspartate, ketamine allows local anesthesia^19^ and exerts an analgesic effect at low dose.^20^ Pre-injection of 10 mg ketamine can reduce the incidence rate of injection pain from 84% to 26% and also partially counteract the blood pressure-reducing effect of propofol.^21^

The injection pain of propofol is manifested as immediate pain or delayed pain (delayed by 10-20s).^22^ To prevent the immediate pain, ketamine was injected 30s before propofol injection. Pre-injecting or mixing low-dose ketamine both decreased the incidence rate of injection pain, exceeding the outcomes using 0.2 mg/kg lidocaine pre-injection. Meanwhile, the hemodynamics was not obviously affected. Nevertheless, ketamine has well-documented side effects such as delayed recovery, postoperative nausea and vomiting, and mental excitement.^23^ In our study, the adverse reactions of all groups were not significantly different, which may be ascribed to the low dose of ketamine and the small sample size.

Given that pain is affected by various subjective factors, it is crucial to stabilize the psychological states of patients by establishing a quiet, peaceful environment, communicating with them, allowing family companion and toy playing, and relieving their anxiety to the maximum extent.^24^ Though we had endeavored to eliminate the above factors, subjective backgrounds such as mental health, degree of awareness and pain tolerance could not be determined based on fixed criteria, so the experimental results may be affected.

To relieve the injection pain of propofol, researchers have been devoted to the following two aspects. First is the combination of several drugs.^25^ For instance, Zhang et al. evaluated the pain on injection of propofol via different combinations of fentanyl, sufentanil or remifentanil in gastrointestinal endoscopy.^26^ They found that propofol and sufentanil group was the most suitable program for painless gastroscopy. Second is the use of M/LCT as the solvent. West et al. compared and systematically assessed the effects of several anesthesia methods, and reported that using M/LCT effectively mitigated the injection pain of propofol.^9^ In this study, we tested the effects of both drug combination and M/LCT.

In summary, the injection pain of propofol was significantly alleviated by using M/LCT, pre-injecting 0.5 mg/kg lidocaine or 0.2 mg/kg ketamine, or pre-mixing 180 mg propofol with 40 mg lidocaine or 16 mg ketamine, with significant differences from those of the control group treated by normal saline (P<0.01). Hence, they are potentially effective strategies for preventing the injection pain of propofol. However, the dose and combination of these anesthetics should be further studied to eliminate such pain.

### Authors’ contributions

**DC & LL:** Data collection and analysis.

**ZH:** Study design and manuscript writing.
